# Nasal Rifampicin Halts the Progression of Tauopathy by Inhibiting Tau Oligomer Propagation in Alzheimer Brain Extract-Injected Mice

**DOI:** 10.3390/biomedicines10020297

**Published:** 2022-01-27

**Authors:** Tomohiro Umeda, Rumi Uekado, Keiko Shigemori, Hiroshi Eguchi, Takami Tomiyama

**Affiliations:** 1 Department of Translational Neuroscience, Graduate School of Medicine, Osaka City University, 1-4-3 Asahimachi, Abeno-ku, Osaka 545-8585, Japan; umemaru@med.osaka-cu.ac.jp (T.U.); m8589781@med.osaka-cu.ac.jp (R.U.); d20ma003@eb.osaka-cu.ac.jp (K.S.); 2 Pharmaceutical Discovery Research Laboratories, Teijin Pharma Ltd., 4-3-2 Asahigaoka, Hino 191-8512, Japan; h.eguchi@teijin.co.jp

**Keywords:** rifampicin, intranasal administration, tau oligomer, propagation, Alzheimer’s disease, frontotemporal lobar degeneration, tauopathy

## Abstract

The cell-to-cell transmission of tau aggregates is considered a mechanism underlying the intracerebral spreading of tau pathology in Alzheimer’s disease (AD) and other tauopathies. Recent studies suggest that tau oligomers, rather than fibrils, participate in this process. We previously showed that intranasal rifampicin inhibits tau oligomer accumulation and improves cognition in tauopathy mice. In the present study, we examined the effects of nasal rifampicin on tau propagation in a new mouse model of tauopathy. A tau oligomer-rich fraction prepared from the brain of an AD patient was injected into a unilateral hippocampus of tau264 mice that express both 3-repeat and 4-repeat wild-type human tau. Rifampicin administration was started one week after the injection and performed three times a week for 24 weeks. Cognitive function and tau pathology were assessed by the Morris water maze test and brain section staining. Rifampicin treatment inhibited the spreading of tau oligomers from the injection site to other brain regions and neurofibrillary tangle formation in the entorhinal cortex. Synapse and neuronal loss in the hippocampus were also prevented, and cognitive function remained normal. These results suggest that intranasal rifampicin could be a promising remedy that halts the progression of tauopathy by inhibiting tau oligomer propagation.

## 1. Introduction

Neurodegenerative diseases with a cerebral accumulation of tau aggregates are called tauopathies and include Alzheimer’s disease (AD) and frontotemporal lobar degeneration (FTLD) such as Pick’s disease (PiD), progressive supranuclear palsy (PSP), corticobasal degeneration (CBD), and frontotemporal dementia with Parkinsonism linked to chromosome 17 (FTDP-17) [1,2,3]. In these disorders, the tau pathology arises in a certain brain region and spreads throughout the brain as the disease progresses [3]. For example, in AD, neurofibrillary tangles (NFTs) first appear in the transentorhinal region and spread to the entorhinal cortex, hippocampus, and then cerebral cortex [3,4]. Accumulating evidence suggests that this intracerebral spreading of tau pathology is mediated by the cell-to-cell transmission of tau aggregates [1,2,3,5]. Tau aggregates are thought to be secreted from degenerating neurons into the extracellular space by exocytosis or in exosomes/ectosomes and taken up by the connecting neurons by receptor-dependent/independent endocytosis or membrane fusion between the exosomes/ectosomes and the plasma membrane. Alternatively, tau aggregates may be transported to neighboring neurons through tunneling nanotubes like prions. The internalized tau aggregates act as a seed for subsequent de novo aggregation by normal tau expressed in the recipient cells. Thus, preventing the cell-to-cell transmission of tau aggregates could be effective at halting the disease progression, suggesting tau-sequestering medicines, such as anti-tau antibodies, should target extracellular tau aggregates [3].

There are six isoforms of tau expressed in an adult human brain; three of them possess three repeats (3R) of the microtubule-binding domain, and the others have four repeats (4R). These isoforms are produced from a single gene (*MAPT*) on chromosome 17 by alternative splicing of exons 2, 3 and 10. The exon 10 codes the second microtubule-binding domain. It has been shown that both the exonic and intronic mutations in the *MAPT* gene identified in FTDP-17 affect the splicing of tau mRNA and/or aggregation property of tau protein [1]. No *MAPT* mutations have been reported in AD. In AD and some cases of FTDP-17, all six isoforms participate in the NFT formation [1,2,3]. In contrast, tau inclusions formed in PiD contain only 3R tau isoforms, while those in PSP and CBD are composed of only 4R tau isoforms [1,2,3]. It is unclear why only 3R or 4R tau isoforms selectively aggregate in those diseases. Unlike humans, mice express only 4R tau isoforms at adult age [6]. Thus, wild-type mice may not be an adequate model for the study of human tauopathy, although some experiments have been done by injecting human brain extracts into the brain of wild-type mice [7]. In many cases, tau propagation experiments are performed using human tau transgenic mice, which mostly express 4R human tau [8,9,10]. In these mice, tau propagation is mediated by 4R human/mouse tau, even though the mixture of 3R and 4R tau is used as a seed. Previously we generated a new tau transgenic line, tau264, which expresses both 3R and 4R human tau [11]. These mice do not show any pathology or abnormal behavior even at 24 months. Thus, tau264 mice are expected to be a good tool to study tau propagation mediated by both 3R and 4R tau.

The nature of tau aggregates involved in the propagation is exactly unknown. It is believed that tau species responsible for the propagation are large, insoluble fibrillar aggregates [9,10,12]. However, recent studies suggest that the true culprit causing the pathological alterations are relatively small, soluble oligomers of tau [13,14]. Tau oligomers have also been shown to be secreted from neurons and transmitted to neighboring cells to induce tau pathology [15,16,17,18]. We previously showed that a well-known antibiotic, rifampicin, inhibits the oligomer formation of Aβ, tau, and α-synuclein in vitro, and when administered orally or intranasally improves cognition by reducing those amyloidogenic protein oligomers in the brains of mouse models of AD, FTLD-tau, and dementia with Lewy bodies, respectively [19,20,21]. These findings imply that rifampicin could prevent the propagation of tau oligomers and thereby halt the disease progression. Thus, in the present study, we examined the effects of nasal rifampicin on tau pathology spreading and cognitive decline in a new mouse model of tau propagation using tau264 mice.

## 2. Materials and Methods

### 2.1. Preparation of Inoculum

Previously, we examined several brain samples obtained by the autopsies of AD patients for tau pathology by Western blot [22]. In the present study, we chose one sample (female, 80 years old, sporadic AD) from those stocks to prepare the inoculum. The brain tissue (5.0 g) was homogenized in 30 mL of artificial cerebrospinal fluid (aCSF; ARTCEREB, Otsuka Pharmaceutical, Tokyo, Japan). The homogenate (Hg) was centrifuged at 27,000× *g* at 4 °C for 20 min to remove insoluble materials. The resultant supernatant (S1) was centrifuged again at 150,000× *g* at 4 °C for 20 min to separate soluble (S2) and insoluble fractions (P2). Each fraction was dispensed into tubes and frozen at −80 °C until use. The Hg, S1, S2, and P2 fractions were subjected to Western blot with an anti-tau antibody, pool-2 [6], followed by horseradish peroxidase (HRP)-labelled second antibodies (Bio-Rad Laboratories, Hercules, CA, USA) and chemiluminescent HRP substrate (ImmunoStar LD; Fujifilm-Wako, Osaka, Japan). The stained tau species were visualized using an ImageQuant LAS 500 image analyzer (GE Healthcare, Hino, Japan). The data showed that tau oligomers were preferentially collected in P2 fraction. Therefore, we decided to use this fraction as an inoculum hereafter.

### 2.2. Intracerebral Injection and Intranasal Rifampicin Treatment

Eight-month-old tau264 mice were used. These mice express both 3R and 4R wild-type human tau (tau410 and tau441, respectively) at a ratio of 1:1 under the mouse CaMKIIa promoter and do not show any pathology or abnormal behavior even at 24 months [11]. The mice were divided into 3 groups: one for P2 injection and rifampicin treatment (*n* = 11: 6 male and 5 female), one for P2 injection and vehicle treatment (*n* = 14: 7 male and 7 female), and one for sham-operated aCSF injection and vehicle treatment (*n* = 11: 5 male and 6 female). One microliter of P2 fraction or aCSF was injected into a unilateral hippocampus at a position of −2.5 mm rostral, −2.5 mm lateral, and −2.3 mm ventral from the bregma. Three days after the injection, other mice that received the P2 injection were sacrificed and examined for tau pathology in the injection site. Rifampicin treatment was started 1 week after the injection. Rifampicin (Sigma-Aldrich, St. Luis, MO, USA) was dissolved to 10 mg/mL in 0.5% low-viscosity carboxymethylcellulose (CMC, Sigma-Aldrich), and 10 μL of the solution was administered intranasally 3 times a week for 24 weeks. Control groups received CMC for 24 weeks. After the treatment, mouse cognition was assessed by the Morris water maze. Then, mouse brain sections were prepared and examined for the spreading of tau pathology.

### 2.3. Behavioral Tests

Following the 24-week rifampicin treatment, the cognitive function of the mice was evaluated by the Morris water maze test as described previously [22]. As a normal control, age-matched non-transgenic littermates (*n* = 11: 5 male and 6 female) were also tested. Mice were trained to swim to a hidden platform 5 times a day at 5-min intervals over 4 consecutive days. The time when the mice climbed on the platform was recorded. The mean time of the 5 trials was calculated each day. Rifampicin treatment was continued during the behavioral test. At day 5, the retention of spatial reference memory was assessed by a probe trial consisting of a 60-s free swim in the pool without the platform.

### 2.4. Histological Analysis

After the water maze task, each group was divided into two groups: one group for immunohistochemical analysis and the other for biochemical analysis. Brain sections were prepared as described previously [23]. To expose the antigens, sections were boiled in 0.01 N HCl, pH2 for 10 min (for 3R and 4R tau) or 10 mM citrate buffer, pH6 for 30 min (for phosphorylated tau, tau oligomers, synaptophysin, NeuN, and Iba1). After blocking with 10% calf serum overnight, the sections were stained with antibodies for pSer202/Thr205-tau (AT8, Thermo Scientific, Waltham, MA, USA), tau oligomers (TOMA1; Merck-Millipore, Darmstadt, Germany), 3R tau (RD3; Merck-Millipore), 4R tau (R2; a rabbit antibody developed in our lab) [6], synaptophysin (SVP-38; Sigma-Aldrich), NeuN (Chemicon, Temecula, CA, USA), and Iba1 (Fujifilm-Wako), essentially as described previously [24]. The staining was followed by biotin-labelled second antibody (Vector Laboratories, Burlingame, CA, USA), HRP-conjugated avidin-biotin complex (Vector Laboratories), and a HRP substrate, diaminobenzidine (DAB; Dojindo, Kumamoto, Japan). For the immunofluorescence imaging, FITC- or rhodamine-labelled second antibody (Jackson Laboratory) was used. For the detection of NFTs, Gallyas silver staining was performed as described previously [11]. As a normal control for the synaptophysin and NeuN staining, brain sections of age-matched non-transgenic littermates were stained. The stained specimens were viewed under a BZ-X800 fluorescence microscope (Keyence, Osaka, Japan), and the images were collected. Phosphorylated tau, tau oligomers, and synapse loss were evaluated by quantifying the staining intensity in a constant area in each image using NIH ImageJ software. NFT formation and neuronal loss were estimated by counting the silver-positive and NeuN-positive cells, respectively, in a constant area in each image.

### 2.5. Western Blot Analysis of Propagated Tau

Brains were collected from each group (*n* = 5–6) after the water maze task. Contralateral cerebral cortices were homogenized by sonication in 5 volumes of Tris-buffered saline containing a protease inhibitor cocktail (P8340; Sigma-Aldrich) and a phosphatase inhibitor cocktail (Nacalai Tesque, Kyoto, Japan). The homogenates were subjected to Western blot with an anti-tau antibody, pool-2.

### 2.6. Statistical Analysis

All experiments and data analyses were performed under unblinded conditions. Comparisons of means among more than two groups were performed using analysis of variance (ANOVA) or two-factor repeated measures ANOVA (for the Morris water maze acquisition test), followed by Fisher’s PLSD test. Differences with a *p*-value of <0.05 were considered significant.

## 3. Results

**Figure 1:**
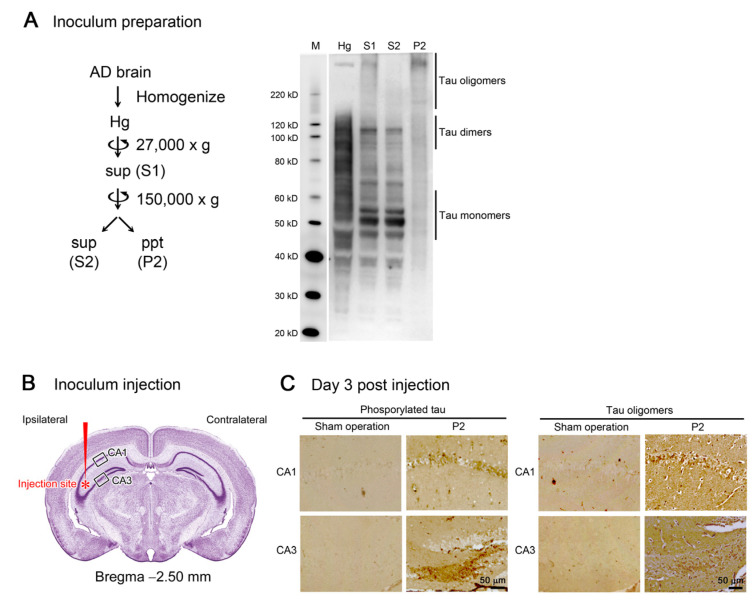
Preparation and injection of tau oligomer-rich inoculum. (**A**) AD brain homogenate (Hg) was centrifuged to remove insoluble materials. The supernatant (S1) was further separated by ultracentrifugation into soluble (S2) and insoluble (P2) fractions. In Western blot analysis, tau monomers and dimers were primarily collected in S2 fraction, whereas tau oligomers more than 120 kD were rich in P2 fraction. M, MagicMark XP Western Protein Standard (Invitrogen, Carlsbad, CA, USA). (**B**) P2 fraction was injected into a unilateral hippocampus of 8-month-old tau264 mice. (**C**) Three days after the injection, brain sections were examined for phosphorylated and oligomeric tau species. These pathogenic molecules were detected within cells in the injection site, indicating their successful internalization. Intranasal rifampicin treatment was started 1 week after the injection.

Initially, we prepared the inoculum to be used in the tau propagation experiments. Brain tissues from an AD patient were homogenized in aCSF and centrifuged at 27,000× *g* to remove large insoluble materials. The resultant supernatant (S1) was centrifuged again at 150,000× *g* to separate the soluble (S2) and insoluble (P2) fractions. Western blot analysis revealed that S2 fraction primarily consists of tau monomers and dimers, whereas P2 fraction dominantly contains tau oligomers with molecular sizes more than 120 kD (Figure 1A). Thus, we decided to use P2 fraction hereafter as the tau oligomer-rich inoculum.

**Figure 2:**
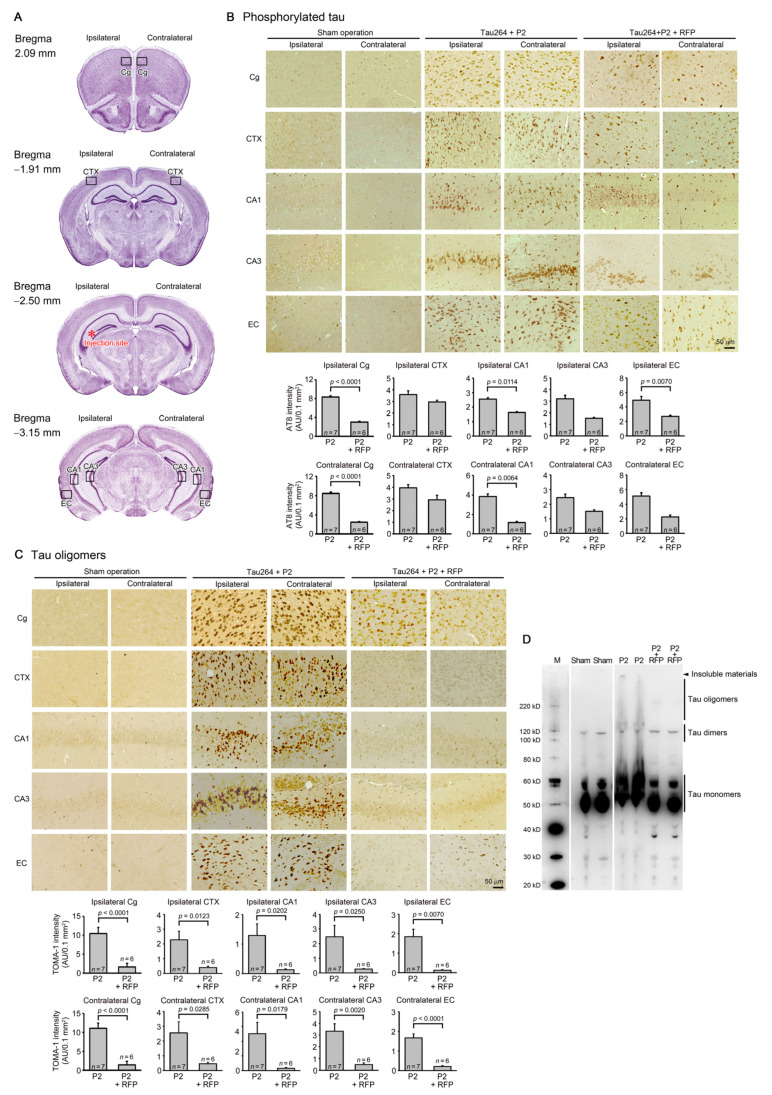
Intracerebral spreading of phosphorylated and oligomeric tau species and the effects of rifampicin treatment. (**A**) After 24 weeks of rifampicin treatment, brain sections anterior and posterior to the injection site were examined for the spreading of tau pathology. Cg, cingulate cortex; CTX, cerebral cortex; CA1 and CA3, hippocampal CA1 and CA3 regions; EC, entorhinal cortex. (**B**) In P2-injected, CMC-treated mice, AT8-positive phosphorylated tau was detected in all regions analyzed, while sham-operated mice did not show any pathology. Rifampicin (RFP) treatment significantly prevented phospho-tau spreading into the cingulate cortex, hippocampal CA1 regions, and entorhinal cortex. (**C**) In P2-injected, CMC-treated mice, TOMA1-positive tau oligomers were detected in all regions analyzed, while sham-operated mice did not show any pathology. Rifampicin treatment prevented oligomer spreading almost completely except for the cingulate cortex. (**D**) In Western blot analysis, the accumulation of tau oligomers with molecular sizes more than 120 kD was observed in the contralateral cerebral cortices of P2-injected, CMC-treated mice. Insoluble materials were also detected in these mice (arrowhead). Rifampicin treatment attenuated these tau aggregates. M, MagicMark standard.

**Figure 3:**
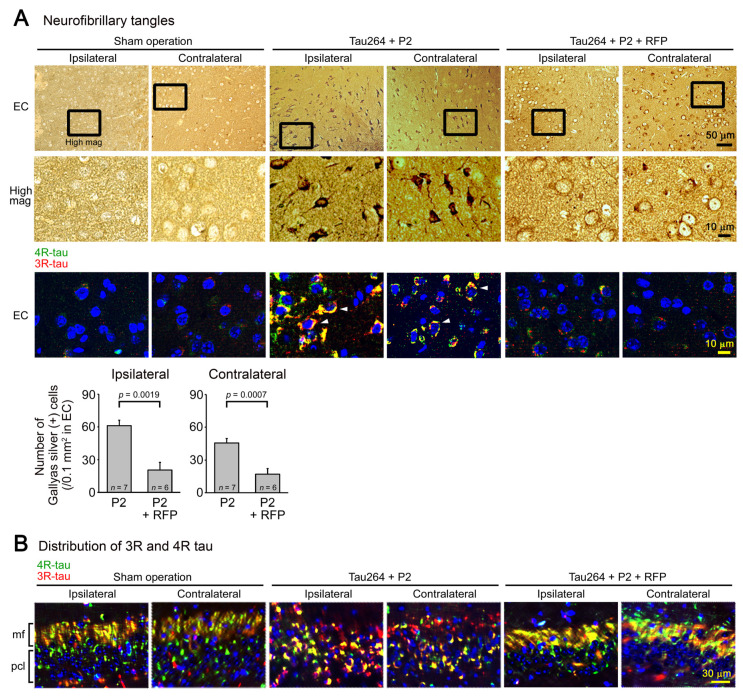
NFT formation and the effects of rifampicin treatment. (**A**) Gallyas silver staining was performed to visualize NFTs. In P2-injected, CMC-treated mice, NFT-bearing neurons were detected in the entorhinal cortex (EC), but not in other brain regions bilaterally. Some neurons showed a shrunk morphology. Immunofluorescence imaging revealed that in these cells, both 3R- and 4R-tau accumulated within the cell bodies (arrowheads). Sham-operated mice did not show these alterations, and rifampicin treatment prevented them. (**B**) In the hippocampal CA3 region, an altered distribution of 3R and 4R tau was observed in P2-injected, CMC-treated mice but not in sham-operated mice: 4R tau disappeared from the mossy fibers (mf), and both 3R and 4R tau accumulated within the cell bodies in the pyramidal cell layer (pcl). Rifampicin treatment prevented this alteration.

**Figure 4:**
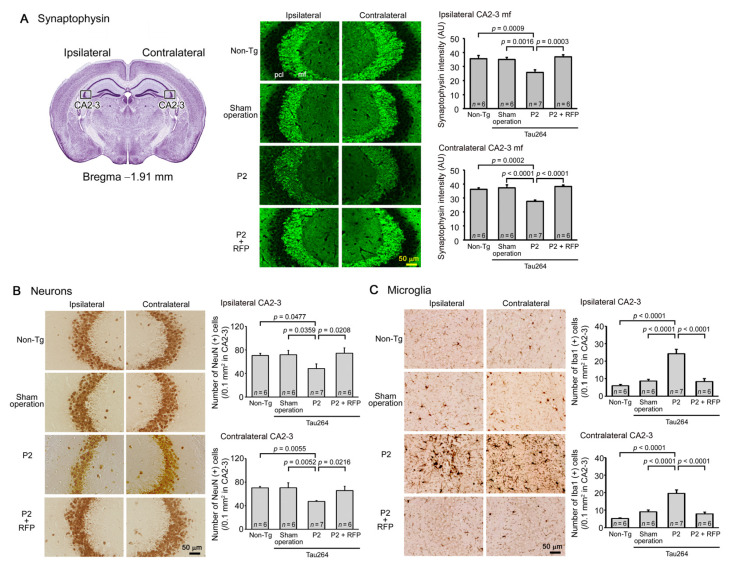
Loss of synapses and neurons and the effects of rifampicin treatment. Brain sections anterior to the injection site were examined for synapse and neuronal loss and microglial activation. (**A**) P2-injected, CMC-treated mice, but not sham-operated mice, showed a significant decrease in synaptophysin levels in the hippocampal mossy fibers bilaterally. Rifampicin treatment prevented this decrease. (**B**) P2-injected, CMC-treated mice, but not sham-operated mice, showed a significant decrease in NeuN-positive cells in the hippocampal CA2-3 region bilaterally. Again, rifampicin treatment prevented this decrease. (**C**) P2-injected, CMC-treated mice, but not sham-operated mice, showed a significant increase in Iba1-positive microglia in the hippocampal CA2-3 region bilaterally. Rifampicin treatment prevented this inflammatory reaction. Non-Tg, Non-transgenic.

One microliter of the inoculum was injected into a unilateral hippocampus of 8-month-old tau264 mice (Figure 1B). At day 3, phosphorylated tau and tau oligomers were detected within cells in and around the injection site (Figure 1C), indicating that the injected pathological tau species were successfully internalized into the cells. One week after the injection, rifampicin treatment was started. Rifampicin (0.1 mg in 10 μL CMC) was administered intranasally three times a week for 24 weeks. After the treatment, brain sections were prepared and examined for the spreading of tau pathology. The regions we analyzed include the cingulate cortex and cerebral cortex anterior to the injection site, and the hippocampal CA1 and CA3 regions and entorhinal cortex posterior to the injection site in both the ipsilateral and contralateral sides (Figure 2A), all of which have neural connections with the injection site [25]. For P2-injected, CMC-treated mice, AT8-positive phosphorylated tau was detected in all the regions bilaterally, but sham-operated mice did not show any pathology (Figure 2B). Rifampicin treatment inhibited the spreading of phospho-tau and significantly reduced the levels in the cingulate cortex, hippocampal CA1 regions, and entorhinal cortex. TOMA1-positive tau oligomers were also detected in all regions in the P2-injected, CMC-treated mice, but, again, sham-operated mice did not show any pathology (Figure 2C). Rifampicin treatment inhibited tau oligomer spreading almost completely except for the cingulate cortex. Western blot analysis revealed that P2 injection induced the accumulation of tau oligomers with molecular sizes more than 120 kD in the contralateral cerebral cortices (Figure 2D). In addition, insoluble materials were detected on the gel top suggesting the NFT formation in these mice. Rifampicin treatment attenuated these tau aggregates. Furthermore, molecular sizes of tau monomers were slightly decreased by rifampicin treatment indicating that rifampicin prevented the increase of phospho-tau. We performed Gallyas silver staining to visualize NFTs. In the P2-injected, CMC-treated mice, NFTs were abundantly detected in the entorhinal cortex bilaterally, but not in other brain regions (Figure 3A). Some silver-positive neurons showed a shrunk morphology, suggesting neurodegeneration. Immunofluorescence imaging revealed that both 3R and 4R tau isoforms participated in the NFT formation (arrowheads in Figure 3A). Rifampicin treatment prevented the intracellular accumulation of 3R and 4R tau and significantly reduced the number of silver-positive cells. In the hippocampal CA3 region, both 3R and 4R tau dominantly localized to the mossy fibers, but not neuronal cell bodies in sham-operated mice (Figure 3B). P2 injection caused an abnormal distribution of tau isoforms: 4R tau disappeared from the mossy fibers, and both 3R and 4R tau accumulated in neuronal cell bodies. This alteration is very similar to that previously reported in tau264 × APP_OSK_ double transgenic mice [24] and may be a sign of pre-tangle formation. Rifampicin treatment prevented this pathological change. We further examined synapse and neuronal loss. In the hippocampal CA2-3 regions, the intensity of synaptophysin in the mossy fibers was significantly reduced by P2 injection in both the ipsilateral and contralateral sides (Figure 4A). Rifampicin maintained synapses levels similar to those in non-transgenic littermates and sham-operated tau264 mice. In the same regions, a significant decrease in the number of NeuN-positive cells was observed in both sides of P2-injected mice (Figure 4B). Again, rifampicin protected neurons from the degeneration. We also evaluated microglial activation by staining with an antibody to Iba1. P2 injection caused a significant increase in Iba1-positive cells in the hippocampal CA2-3 regions bilaterally. Rifampicin treatment prevented this inflammatory reaction (Figure 4C).

**Figure 5:**
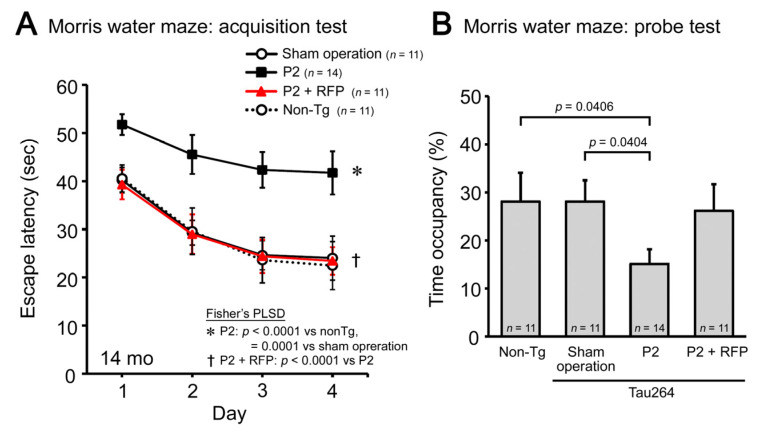
Cognitive decline and the effects of rifampicin treatment. The cognitive function of mice was evaluated by the Morris water maze. (**A**) P2-injected, CMC-treated mice, but not sham-operated mice, showed a significant impairment in the acquisition of spatial reference memory. This cognitive decline was prevented by rifampicin treatment. (**B**) In the probe trials, P2-injected, CMC-treated mice showed disturbed memory retention. Rifampicin-treated mice possessed normal retention.

Finally, we evaluated the cognitive function of mice by the Morris water maze. P2 injection caused a significant impairment in both the acquisition (Figure 5A) and retention of spatial reference memory (Figure 5B). Rifampicin prevented the cognitive decline, and the treated mice showed a cognition similar to non-transgenic littermates and sham-operated tau264 mice. These results suggest that intranasal rifampicin has therapeutic potential to inhibit the progression of tauopathy by halting tau oligomer propagation.

## 4. Discussion

In the present study, we generated a new mouse model of tau propagation using tau264 mice. Tau264 mice express both 3R and 4R human tau without showing any pathology or abnormal behavior even at 24 months [11]. We prepared a tau oligomer-rich fraction from a human AD brain and injected it into a unilateral hippocampus of tau264 mice. During 24 weeks after the injection, phosphorylated tau and tau oligomer pathologies spread from the injection site to other brain regions including the bilateral cingulate cortex, cerebral cortex, hippocampus, and entorhinal cortex. These regions are connected by a neural network [25]. The entorhinal cortex displayed abundant NFTs, which were composed of both 3R and 4R tau isoforms like NFTs in AD [1,2,3]. Furthermore, those mice exhibited synapse and neuronal loss and eventually cognitive decline. These features indicate that this model reproduces well the phenotype of tau pathology in AD. It has been shown that seed-induced spreading of tau pathology occurs along neuronal connections to impair network function and associated behaviors and that the propagation is mediated by tau oligomers [26]. This effect seems to be in good agreement with our results. We observed an abnormal distribution of 3R and 4R tau that accumulated in neuronal cell bodies. This distribution might have occurred because the secreted tau oligomers transmitted the synapses to enter the postsynaptic cell bodies in which the internalized oligomers recruited normal tau from the axon to form aggregates [13]. Furthermore, we observed neuronal loss in the hippocampal CA2-3 regions where NFT formation was not detected. This observation suggests that the real toxic tau species is relatively small oligomers rather than insoluble large fibrils. Since we used an extract of AD brain as the inoculum, which contains not only tau aggregates, but also Aβ pathological species, the initial pathological alteration observed in the injection site might have been triggered by both tau and Aβ. However, the subsequent spreading of tau pathology in tau264 mice should be mediated only by endogenous tau proteins in the absence of human Aβ expression. In conventional transgenic mice that only express either 3R or 4R human tau, tau inclusions are formed with either 3R or 4R tau, like Pick bodies in PiD or tau inclusions in CBD/PSP [1,2,3]. In contrast, tau264 mice, which express both 3R and 4R human tau, can reproduce all types of tau pathology depending on the inoculum. This is an advantage of tau264 mice in studying the pathogenesis of human tauopathy. Very recently, a novel tau propagation model has been reported using genome-edited mice that express both 3R and 4R mouse tau even at adult age [27]. This mouse model has been shown to develop characteristic tau pathologies depending on the inoculum: AD, PiD, or CBD brain extracts.

Using our new mouse model, we studied the effects of nasal rifampicin on tau pathology spreading and cognitive decline. Intranasal rifampicin treatment, which was started 1 week after the seed injection, repressed the spreading of phosphorylated and oligomeric tau species from the injection site to other brain regions in both sides. Consequently, NFT formation, synapse loss, neuronal loss, microglial activation, and cognitive decline were prevented. The mechanism by which rifampicin inhibited tau propagation is unclear. Given that rifampicin treatment was started 1 week after the injection, at which time pathological tau species in the inoculum were already internalized into the cells, it is unlikely that rifampicin prevented tau propagation because rifampicin detoxified the inoculum. Our previous study indicates that although rifampicin binds to oligomers of amyloidogenic proteins, i.e., Aβ, tau, and α-synuclein, to dissociate them into monomers, the levels of preformed amyloid plaques in mouse brain appeared not to change [19]. It is likely then that rifampicin prevented tau propagation by interacting with tau oligomers that were released from degenerating neurons into the extracellular space and promoting their dissociation into monomers. Supporting this hypothesis was our observations that the effects of rifampicin were displayed preferentially against tau oligomers rather than phospho-tau (Figure 2B,C). Since rifampicin can enter the cells, intracellular tau oligomers could be a target of rifampicin too. However, it is unclear whether rifampicin can dissociate tau fibrillar aggregates into monomers. Considering that rifampicin has a tropism to amyloidogenic protein oligomers [19], our results imply that the pathological tau species responsible for the propagation is soluble oligomers and not insoluble fibrils.

**Figure 6:**
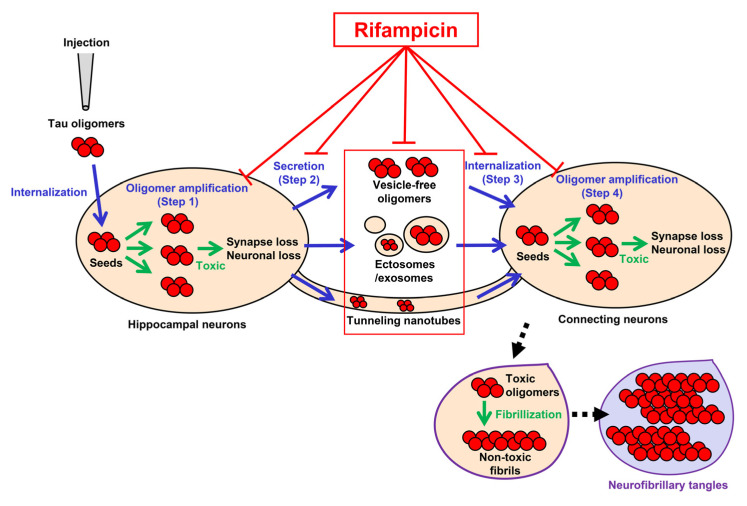
Pathways of tau oligomer propagation and the actions of rifampicin. Injected tau oligomers are internalized to the hippocampal neurons and act as seeds for oligomer amplification (green arrows in step 1). The newly generated tau oligomers are secreted from the cells into the extracellular space as vesicle-free oligomers or in ectosomes/exosomes (step 2). The secreted tau oligomers are internalized to the connecting neurons (step 3). Alternatively, tau oligomers may be transported to neighboring neurons through tunneling nanotubes. The internalized tau oligomers act as seeds for de novo oligomer amplification (green arrows in step 4). Rifampicin has the potential to interfere with all the steps (T-shaped red lines). Tau oligomers are toxic and cause synapse and neuronal loss, while insoluble tau fibrils in neurofibrillary tangles are probably non-toxic. Blue arrows indicate the transmission of tau oligomers. Black arrows with dashed line indicate the process of NFT formation.

The tau propagation hypothesis provides several treatment strategies. The cell-to-cell transmission process of tau aggregates consists of (1) the formation of transmittable tau aggregates in the donor cells, (2) the secretion of the aggregates into the extracellular space, (3) the internalization of the aggregates into the connecting cells, and (4) the seed-dependent aggregation of normal tau expressed in the recipient cells [1,2,3,5] (Figure 6). Step 1 and step 4 can be suppressed by tau aggregation inhibitors or detoxifiers if these compounds enter the cells [28,29]. Step 2 might involve the autophagy–lysosome pathway, suggesting autophagy activators can prevent the release of tau aggregates [30]. Extracellular tau aggregates in step 2 and step 3 can also be a target of anti-tau antibodies in immunotherapy [3,31]. Step 3 might be mediated by cell surface molecules, such as heparan sulfate proteoglycan [1,2,3,5], and, if so, their blockers could disturb the internalization of tau aggregates [32]. However, such antibodies and blockers would be ineffective if tau transmission occurs in exosomes/ectosomes or through tunneling nanotubes. In contrast, rifampicin has the potential to interfere with all the steps by binding to intracellular and extracellular tau oligomers to inhibit their binding to the cell surface [33] and dissociate them into monomers [19] along with restoring autophagy activity [19]. In another report, we confirmed that by combining rifampicin and resveratrol, autophagy-like cellular clearance activity was remarkably enhanced [34]. Taken together with our previous findings, the present results suggest that rifampicin is effective at halting not only the onset, but also the progression of tauopathy.

As we focused on the effects of rifampicin on tau propagation, we used tau264 mice in the present study. However, in AD, both tau and Aβ pathologies spread throughout the brain interacting with each other. To correctly understand the effects of rifampicin on the spreading of AD pathologies, it may be desirable to use model animals that express both human tau and human Aβ. In addition, in human patients, tau and Aβ oligomers are constantly produced, which may require long-term drug administration for months and years. Although nasal administration of rifampicin is safer than oral administration [20], such long-term treatment with rifampicin may still cause adverse effects such as liver dysfunction and drug-drug interaction (Drugs.com website: https://www.drugs.com/mtm/rifampin.html, accessed date is 19 December 2021). As a solution to this problem, we have previously devised a new formula of rifampicin. Simultaneous administration of rifampicin and resveratrol completely eliminated the toxicity of rifampicin and furthermore significantly increased its therapeutic efficacy [34]. The long-term safety of this combinatorial medicine remains to be tested. We observed that microglial activation occurred in parallel with tau pathology spreading. This may have been caused by microglia phagocytosing extracellular tau oligomers. Activated microglia have been shown to release pro-inflammatory cytokines such as IL-1β, IL-6, and TNFα, which may exacerbate tau pathology and lead to neuronal loss [35,36]. Investigating what cytokines are involved is for further study.

Finally, cell-to-cell transmissions are becoming a consensus among researchers as a common mechanism underlying the intracerebral spreading of the pathology of several neurodegenerative diseases [5,37,38]. Considering the broad spectrum of rifampicin against various amyloidogenic proteins [19,34], intranasal rifampicin and resveratrol combination could be a promising remedy for the treatment of those disorders.

## Data Availability

The data presented in this study are available upon request.
